# Annotations

**Published:** 1887-01-22

**Authors:** 


### Annotations.

Is Alcohol a Food?

Dr. J. A. Gaven writes to *The Times* to explain his part in the case in which a man was disallowed the benefits of the lodge of "Total Abstinent Sons of the Phœnix," because he took alcohol according to medical orders. The judge, unfortunately, on a legal point, felt bound to decide in favour of the lodge. Tennyson exhorts to "cut prejudice against the grain." That is not the fashion in these times, but perhaps it ought to be. Total abstinence and its supporters deserve well, on the whole, of the public; but they are terribly ignorant on some important points, and unhappily they have sometimes very one-sided leaders. Is alcohol ever of use as a medicine or as a food? This question will be answered in the most contradictory manner by men who will be ranked as the highest medical and scientific authorities. That being so, what is a mere outsider to do? He must in this, as in other instances, take the general sense of mankind of all ages. That we believe to be a much more trustworthy guide in matters of this kind than the most solemn pronouncements of any individual man, whatever be his medical or his scientific eminence. When asked if alcohol is of any use as a medicine or a food? the plain man may well answer, "I don't know, and if I follow living authorities, it is impossible for me to find out; for whilst the eminent Dr. Smith says it is, the equally eminent Dr. Brown says it is not." The plain man will therefore do well to remember that most men in all countries and at all times have regarded alcohol as *both a medicine and a food.*

Word-puzzles and Gambling.

Our word-puzzles have come to an untimely end. Perhaps it was well that they should have done so. "Nemo" writes a clever letter to *The Times*, in which he denounces the word-puzzle as a system of wholesale gambling. We need hardly say that we had no intention of appealing to the gambling spirit in our readers when we instituted the puzzle. Our object was to do a little towards the support of the hospitals. We were assured by many persons that the system paid well, and that a nice little sum might by its means be periodically apportioned to one or other of the most needy and deserving of the charities. Great was our surprise to find, after a three months' trial, that a very considerable deficit was the result of our well-meant efforts. It could not be that there was any want of interest among our readers, or any want of readers to share the interest. We have had to deal with a large and growing circulation, and with many eagerly interested competitors; and yet, in spite of this, the system, honestly worked, has resulted in a clear loss of several pounds. The obvious conclusion, therefore, is this, that those persons who have found it to pay have not worked it honestly, but have merely been baiting hooks for greedy fish for their own profit. We hope the public will be warned by our experience, and have nothing more to say to word-competitions where an entrance-fee is demanded.

A Royal Commission on Hospitals.

Sir Rutherford Alcock, who is supported by *The British Medical Journal*, asks in *The Times* for the appointment of a Royal Commission to consider the position of the London Hospitals. The *Journal* refers to certain articles written by eminent persons, which have appeared lately (presumably those in The Hospital), and speaks of them as more "distinguished for benevolence than originality." Seeing that none of these articles professed to deal in any way with the subject of Sir R. Alcock's letter, we cannot quite understand why the *Journal* should have gone out of its way to refer to them. However, as originality seems to be the order of the day, and *The British Medical Journal* is admitted on all hands to be at the very source and centre of all illumination, we shall doubtless ere long be furnished with suggestions and guidance of such unmistakable wisdom, that Royal Commissions and other methods of inquiry will be found utterly superfluous. The article in the *Journal* does not, unhappily, contain any of the original suggestions which might have been expected, and therefore they cannot be reproduced for the benefit of our readers. That, however, is doubtless a mere inadvertence, the writer probably not having got beyond his preface. The subject is one of the very first importance, and if *The British Medical Journal*, or any other medium of "light and leading," will suggest such a scheme as will commend itself to the judgment of experienced hospital managers and practical philanthropists, the whole world of charity will gladly doff hats and follow in its footsteps.

